# Transcriptional Responses to Bumped Kinase Inhibitor BKI-1708 in *Toxoplasma gondii* and Human Fibroblasts

**DOI:** 10.3390/cells15151324

**Published:** 2026-07-24

**Authors:** Maria Cristina Ferreira de Sousa, Arunasalam Naguleswaran, Kayode K. Ojo, Wesley C. Van Voorhis, Andrew Hemphill

**Affiliations:** 1 Institute of Parasitology, Vetsuisse Faculty, University of Bern, 3012 Bern, Switzerland; 2 Graduate School for Cellular and Biomedical Sciences, University of Bern, 3012 Bern, Switzerland; 3 Institute of Animal Pathology, Vetsuisse Faculty, University of Bern, 3012 Bern, Switzerland; arunasalam.naguleswaran@unibe.ch; 4 Department of Medicine, University of Washington, Seattle, WA 98109-4766, USA; ojo67kk@uw.edu (K.K.O.); wesley@uw.edu (W.C.V.V.)

**Keywords:** anti-parasitic activity, bumped kinase inhibitor, differential gene expression, human fibroblast, mode of action, *Toxoplasma*, transcriptome

## Abstract

**Highlights:**

**What are the main findings?**

- *T. gondii* baryzoites, formed upon exposure to BKI-1708, exhibit transcriptional changes in genes coding for proteins involved in host cell invasion, protein synthesis, RNA metabolism, and stress responses.
- In uninfected fibroblast host cells, BKI-1708 affects transcripts related to metabolism and detoxification, while in *T. gondii*-infected host cells, increased transcription of immune, lysosomal, and glycan degradation pathways was detected.

**What are the implications of the main findings?**

- Transcriptomics suggests that BKI-1708 interferes in essential biological processes in *T. gondii*, while essential host cell functions are largely unaffected.
- BKI-1708 affects other molecular targets besides the two canonical kinase targets CDPK1 and MAPKL1.

**Abstract:**

Bumped kinase inhibitors are safe with promising efficacy against apicomplexan parasites. The 5-aminopyrazole-4-carboxamide BKI-1708 effectively inhibited vertical transmission of *Toxoplasma gondii* and significantly reduced the cerebral parasite loads in experimentally infected pregnant mice. In vitro experiments revealed that exposure of *T. gondii* tachyzoites to BKI-1708 induces the formation of intracellular multinucleated complexes called “baryzoites”, exhibiting increased expression of bradyzoite-stage proteins while still displaying classical tachyzoite markers. Differential affinity chromatography of *T. gondii* extracts identified numerous BKI-1708-binding proteins involved in invasion/egress, redox homeostasis, and RNA processing. To understand the transcriptional implications of BKI-1708 treatment on *T. gondii* tachyzoites and human foreskin fibroblast host cells, *T. gondii*-infected host cells, either treated with BKI-1708 or untreated, were subjected to dual RNA-seq analysis. BKI-1708 induced a significant transcriptional remodeling in the parasite, with an enrichment in pathways related to translation, RNA metabolism, and stress responses. In contrast, host–cell transcriptional changes were more limited, with transcripts related to metabolic and detoxification programs upregulated in uninfected fibroblasts, and increased transcription of immune, lysosomal, and glycan degradation pathways in infected fibroblasts. These findings suggest that BKI-1708 modulates the transcriptome in a predominantly parasite-specific manner, disrupting essential biological processes in *T. gondii* while largely preserving host cell function.

## 1. Introduction

*Toxoplasma gondii*, being a widespread and highly prevalent obligate intracellular cyst-forming apicomplexan parasite, is the causative agent of toxoplasmosis [1]. Its heteroxenous life cycle includes sexual reproduction exclusively in definitive hosts belonging to the family Felidae [2,3], while most warm-blooded animals, including birds, rodents, livestock, and humans, serve as intermediate hosts [1,4]. Infection occurs through ingestion of undercooked meat containing tissue cysts or consumption of food or water contaminated with oocysts shed by infected cats [1,5,6]. Within the intermediate hosts, *T. gondii* alternates between two asexual stages: the rapidly replicating tachyzoite responsible for acute infection, and the slowly replicating bradyzoite that persists within tissue cysts and establishes chronic infection [1,7]. Toxoplasmosis is one of the most common global zoonotic diseases, considerably impacting human and veterinary health. While most infections are subclinical, severe disease can occur in immunocompromised individuals or upon congenital infection, leading to neurological damage or fetal loss [8]. In livestock, particularly small ruminants, infection can also cause abortion outbreaks with substantial economic consequences [9,10,11].

Current drug treatments for toxoplasmosis in humans primarily rely on combinations of pyrimethamine with sulfadiazine/clindamycin [12], or on atovaquone [13]. Other treatments during the first trimester of pregnancy include spiramycin or a spiramycin/cotrimoxazole combination. Although these treatments are effective against tachyzoites, they fail to eliminate bradyzoite tissue cysts and are often associated with toxicity and limited clinical efficacy [5,14]. Consequently, the persistence of latent infection presents a major therapeutic challenge.

Interest in bumped kinase inhibitors (BKIs) as apicomplexan-specific antiparasitic agents has been growing for over 15 years [15,16]. BKIs target calcium-dependent protein kinases (CDPKs), a family of ATP-competitive serine/threonine kinases unique to apicomplexans and absent in mammalian hosts. In *T. gondii*, CDPKs play important roles in microneme secretion, facilitating host cell invasion, gliding motility, egress, and cell division (endodyogeny). Unlike mammalian kinases, CDPKs have a distinctive structure comprising an N-terminal kinase domain, a connector domain fused to a C-terminal calmodulin-like (CAD) domain. In the absence of calcium, the catalytic domain of the CDPK enzyme is blocked. However, in the presence of calcium, conformational changes occur, exposing the catalytic site and activating the enzyme [16].

The presence of a small gatekeeper residue, typically a glycine or serine, enables the design of inhibitors that selectively block CDPKs while sparing host enzymes [17]. Numerous studies have demonstrated the in vivo safety and efficacy of BKIs against various apicomplexan parasites [18,19,20], leading to subsequent optimization and characterization of their structure–activity relationships [21]. One of the primary targets of BKIs is calcium-dependent protein kinase 1 (CDPK1), a conserved enzyme in apicomplexan parasites [22]. CDPK1 plays an essential role in calcium-dependent signaling processes including microneme secretion, gliding motility, and host cell invasion [22,23,24]. In addition, another validated BKI-drug target is the mitogen-activated protein kinase-like protein TgMAPKL-1 in *T. gondii* [25]. TgMAPKL-1 is an essential regulator of the parasite cell cycle, controlling centrosome duplication required for the formation of two daughter cells during endodyogeny. Disruption or mutation of TgMAPKL-1 interferes with this process, leading to defects in parasite cell division and impaired proliferation [26]. In fact, treatment with BKIs induces a characteristic schizont-like phenotype in which parasites form multinucleated complexes. These structures arise from continuous nuclear division in the absence of successful completion of cytokinesis, leading to accumulation of multiple newly formed daughter zoites contained in these enlarged complexes, which are also referred to as “baryzoites” (from the Greek *βαρύς*, meaning massive or inert) [27,28]. Thus, baryzoites are a drug-induced multinucleated stage triggered and sustained by drug pressure in vitro and exhibit features of both tachyzoites and bradyzoites. Similar structures have been observed when *T. gondii* tachyzoites were exposed to diclazuril, a compound targeting components of the mitochondrial respiratory chain and dihydrofolate reductase [29], and the estrogen antagonist tamoxifen [30].

BKI-1708 is a 5-aminopyrazole-4-carboxamide-based BKI that effectively inhibits the proliferation of *T. gondii* and other apicomplexans in vitro. Notably, it demonstrated significant efficacy in preventing congenital toxoplasmosis in experimentally infected pregnant mice [31]. Ultrastructural analysis of baryzoites formed upon exposure of *T. gondii* with BKI-1708 at 2.5 µM confirmed the formation of baryzoites that remain trapped intracellularly, with parasites expressing tachyzoite antigens, the bradyzoite marker TgBAG1 as well as distinct cyst wall markers [32]. Analysis of the BKI-1708-treated *T. gondii* proteome showed downregulated expression of ribosomal proteins, transcription and translation factors, and secretory organelle proteins. However, two alveolin-domain filament proteins and one conserved hypothetical protein were found in baryzoites at higher abundance following BKI treatment [32]. This suggests that the molecular consequences of BKI-1708 treatment seem to extend beyond its canonical kinase targets. Differential affinity chromatography of *T. gondii* tachyzoite and host cell lysates on immobilized BKI-1708 revealed that BKI-1708 exhibited prominent RNA-binding capacity. It interacted with proteins involved in RNA/DNA metabolism, cytoskeletal organization, vesicular trafficking, and secretory organelle function [33]. Interference in RNA metabolism or RNA-associated processes may contribute to the mechanism of action of BKI-1708 and baryzoite induction; thus, some of these proteins could constitute secondary molecular targets of BKIs.

Transcriptomic profiling offers a powerful and unbiased approach to study treatment effects and regulatory changes. Therefore, we performed integrated host–parasite RNA sequencing to define the impact of BKI-1708 exposure in *T. gondii* tachyzoites and human foreskin fibroblast (HFF) host cells.

## 2. Materials and Methods

### 2.1. Parasites, Culture Medium, Biochemicals and Compounds

Culture medium was purchased from Gibco-BRL (Zürich, Switzerland), and biochemicals obtained from Sigma (St. Louis, MO, USA). BKI-1708 was originally synthesized in the Department of Biochemistry of the University of Washington, Seattle, WA, USA and scaled up by WuXi Apptec Inc., Wuhan, China, to >98% purity by LC/MS-MS and NMR and shipped as powder stored at room temperature [34]. Stock solutions of 20 mM were prepared in dimethyl-sulfoxide (DMSO) and stored at −20 °C. Human foreskin fibroblasts (HFF; PCS-201-010™, ATCC LGC, Molsheim Cedex, France) were maintained at 37 °C/5% CO_2_ in Dulbecco’s Modified Eagle Medium (DMEM) supplemented with 10% heat-inactivated and sterile filtered fetal calf serum (FCS), and 1% antibiotic–antimycotic solution (50 U of penicillin/mL, and 50 μg streptomycin/mL), and were passaged at least once a week [20]. The strain used in this study was *T. gondii* ME49 (50611™, ATCC LGC), which was cultivated by serial passaging under standard conditions in HFF monolayer cultures at 37 °C/5% CO_2_ as previously described [20]. Briefly, parasites were maintained by serial passage in HFF monolayers twice a week. Infected cultures were scraped, and intracellular parasites were released by mechanical disruption through repeated passage through a 25-gauge needle. The resulting parasite suspension was used to infect fresh HFF monolayers for continuous propagation.

### 2.2. Infection and Drug Treatment

Samples for transcriptomics were prepared as previously done for proteomics [32]. Semi-confluent HFF monolayers were maintained in T75 cell culture flasks and were infected with 1 × 10^7^ TgME49 tachyzoites (freshly egressed from host cells and counted in a Neubauer chamber) or remained uninfected. At the time point 4 h post infection, treatment with 2.5 µM BKI-1708 was initiated; control cultures were not exposed to BKI-1708 but treated with vehicle only. Three biological replicates were used for each condition. All non-treated control cultures (infected or non-infected) were maintained at 37 °C/5% CO_2_ for 3 days, BKI-1708-treated cultures were maintained for 5 days (BKI-1708). Subsequently, infected and non-infected monolayers were removed with a rubber cell scraper and resuspended in PBS followed by centrifugation (15 min, 1000× *g*, 4 °C) [32].

### 2.3. RNA Isolation

Total RNA was extracted using the RNeasy Mini Kit (Qiagen, Hilden, Germany) according to the manufacturer’s instructions with minor modifications. Up to 1 × 10^7^ cells were harvested as a pellet and resuspended in 350 µL of RLT buffer supplemented with 3.5 µL of 2-mercaptoethanol, followed by thorough homogenization. Subsequently, 350 µL of 70% ethanol was added and mixed by pipetting. Up to 700 µL of the resulting lysate, including any precipitate, was applied to a RNeasy Mini spin column placed in a 2 mL collection tube and centrifuged at >8000× *g* for 15 s, and the flow-through was discarded. The column was washed with 350 µL of RW1 buffer and centrifuged at >8000× *g* for 15 s. Genomic DNA was removed by applying 80 µL of DNase I solution (10 µL DNase I stock in 70 µL RDD buffer) directly onto the membrane, followed by incubation at room temperature for 15 min. The column was washed again with 350 µL RW1 buffer, centrifuged, and subsequently washed twice with 500 µL RPE buffer, with the second wash centrifuged for 2 min to ensure complete drying of the membrane. RNA was eluted in 30 µL of RNase-free water by centrifugation at >8000× *g* for 1 min, and the elution step was repeated to maximize RNA recovery.

### 2.4. Library Preparation and Sequencing Parameters

Libraries were prepared using the Universal Plus™ mRNA-Seq library preparation kit with NuQuant^®^ (Tecan, Männedorf, Switzerland), with polyA selected RNAs. Sequencing of cDNA libraries was performed at the Next Generation Sequencing Facility of the University of Bern, using Illumina NextSeq 1000 sequencing systems (Illumina, Berlin, Germany) with 150 bp paired end reads and sequence depths of ~30 million reads per sample. Raw read files are deposited at the European Nucleotide Archives (ENA) (http://www.ebi.ac.uk/ena) (study PRJEB111857, accessible 23 July 2026).

### 2.5. Alignment Strategy (Dual Genome Mapping—Pipeline)

Reads were processed and mapped using the bowtie2 tool available in the Galaxy Interface (https://usegalaxy.org/, accessed on 4 February 2026) with the following default parameters (-D 15 -R 2 -N 0 -L 22 -i S,1,1.15) to *T. gondii* ME49 reference genome (https://toxoDB.org, Genome version 68) and to human genome (Genome Reference Consortium Human Build 38, GRCh38) were mapped separately. In addition, multi-mapping was not allowed so only the single best-read alignment was reported. The feature Counts tool available in the Galaxy platform was used to extract read counts mapped against the feature “mRNA” using GFF annotation files of *T. gondi* and human. Gene-level quantification and differential expression analyses were performed independently for the parasite and host datasets using the DESeq2 package (https://bioconductor.org, release 3.19) available in the Galaxy platform. Benjamini–Hochberg (BH) procedure was used to control the False Discovery Rate (FDR) upon multiple testing and the adjusted *p*-values were calculated. Genes exhibiting a fold change ≥ 2 and a *p*-value < 0.05 were considered significantly differentially expressed. Our supplementary tables provide all the information on *p*-values and adjusted *p*-values for every gene.

### 2.6. Pathway Enrichment

KEGG pathway enrichment analysis was performed using ShinyGO for host cells, which integrates KEGG pathway annotations. For *T. gondii* ME49 Gene Ontology (GO) enrichment analysis was performed using the LAGOS web platform (https://go.princeton.edu, accessed 4 June 2026), which implements the GO:TermFinder/LAGO algorithms to identify statistically overrepresented GO terms (Biological Process, Molecular Function, and Cellular Component) in a given gene list compared to a defined background set.

## 3. Results

### 3.1. Global Transcriptional Changes in Parasite and Host Cells Under BKI-1708 Treatment

Exposure of *T. gondii* tachyzoites grown in HFF to BKI-1708 induces the formation of MNCs, also known as baryzoites, which remain viable for extended periods of time. These baryzoites have been visualized by electron microscopy in different studies [31,32,33], and the changes induced in these parasites are also illustrated using scanning (SEM) and transmission electron microscopy (TEM) in Supplementary Figure S1.

**Figure 1:**
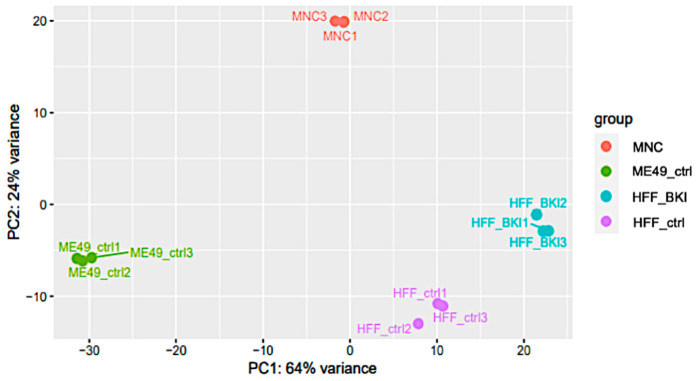
Principal component analysis (PCA) of *T. gondii*-infected host cells with and without BKI-1708 treatment. Each point represents an individual biological replicate. Samples are color-coded by group: non-treated human foreskin fibroblasts (HFF_ctrl) are purple, BKI-treated host cells (HFF_BKI) are blue, non-treated *T. gondii* tachyzoites (ME49_ctrl) are green, and BKI-treated tachyzoites (MNC, “multinucleated complexes”) are red. Clustering reflects both organism type (host versus parasite) and treatments (BKI-treated versus non-treated).

In this study, RNA sequencing (RNA-seq) was performed on *T. gondii* ME49-infected and non-infected HFF, comparing BKI-1708-treated and untreated samples. High-quality RNA was extracted and subjected to Illumina sequencing. Principal component analysis (PCA) showed distinct clustering of host cells and *T. gondii* samples according to treatment conditions (Figure 1). Replicates within each condition are tightly aligned, demonstrating high reproducibility. The first principal component (PC1, 64% variance) separates parasite samples from host cell samples, with untreated *T. gondii* tachyzoites (ME49_ctrl) forming a distinct cluster on the negative axis and BKI-1708-treated tachyzoites (MNC) separating along PC2 (24% variance). Host-derived samples cluster independently, with untreated HFF controls clearly separated from BKI-1708-treated HFF, indicating a transcriptional response to treatment in both host and parasite samples (Figure 1).

**Figure 2:**
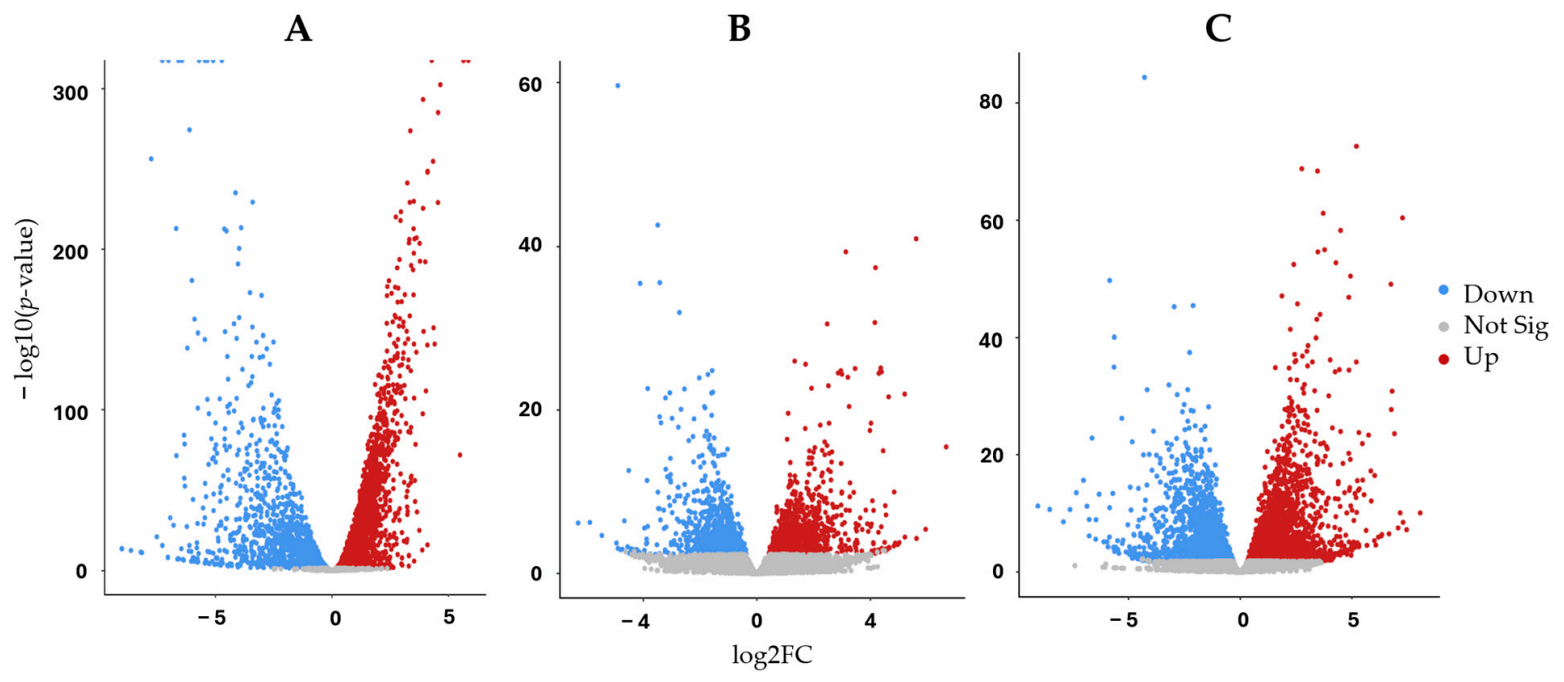
Differential gene expression (DE) analysis by RNA-seq. Volcano plots showing the distribution of differentially expressed genes in (**A**) *T. gondii*, (**B**) non-infected human fibroblasts and (**C**) *T. gondii*-infected human fibroblasts, all treated versus non-treated with BKI-1708. Each point represents a single gene, with the log2 fold change (L2FC) on the *x*-axis and the −log10 adjusted *p*-value on the *y*-axis. Upregulated genes are shown on the right and downregulated genes on the left; significantly upregulated genes are highlighted in red and downregulated genes in blue.

Global transcriptional changes in *T. gondii* tachyzoites, non-infected HFF, and *T. gondii*-infected HFF upon BKI-1708 treatment are summarized in the volcano plots shown in Figure 2. While host cells exhibited modest changes upon infection, the parasite displayed a markedly broader and more pronounced transcriptional reprogramming.

### 3.2. BKI-1708 Induced Transcriptional Alterations in T. gondii

Transcriptomic profiling of treated tachyzoites revealed substantial transcriptional changes in response to treatment. Out of 8140 annotated genes, 1174 (14.4%) corresponding transcripts were upregulated, whereas 878 (10.7%) were downregulated (*p* < 0.05) (Table 1). The percentages of up or downregulated transcripts in infected HFF were substantially lower compared to *T. gondii*, and lowest in non-infected HFF. The entire dataset is accessible in Supplementary Tables S1 and S2.

#### 3.2.1. Parasite Transcriptome Reveals Interference in Biological Pathways upon BKI-1708 Treatment

**Figure 3:**
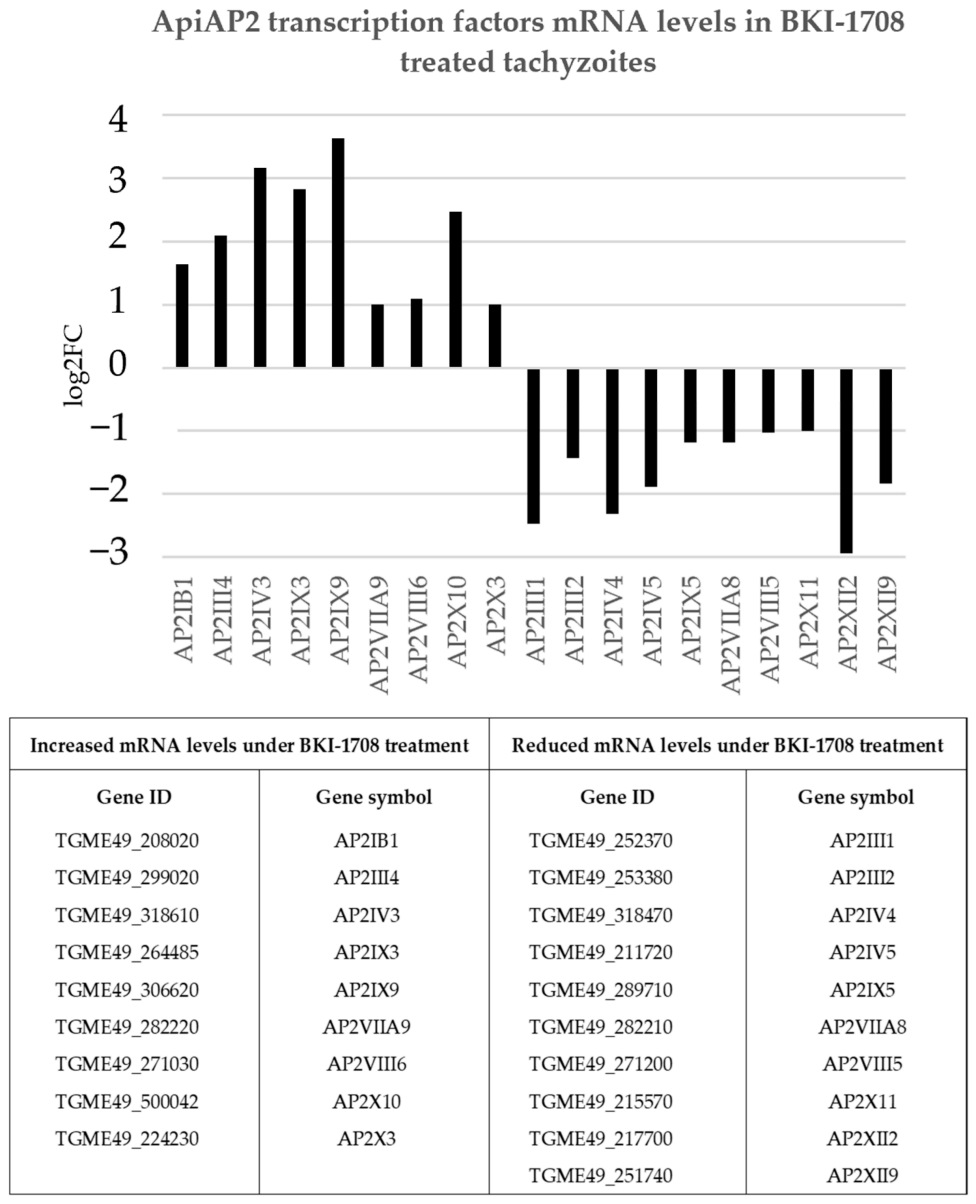
Genes coding for ApiAP2 transcription factors with ≥2-fold decreased/increased expression of mRNA levels upon BKI-1708 treatment compared to untreated tachyzoites. Log2FC = log2-transformed fold changes in mRNA levels derived from DESeq2-normalized RNA-seq read counts.

Treatment with BKI-1708 resulted in transcriptomic remodeling affecting both parasite structural and host–parasite interaction pathways. 37.5% of the downregulated genes were hypothetical proteins. Gene Ontology (GO) analysis of downregulated transcripts identified three pathways namely, inner membrane pellicle complex (GO:0070258), pellicle (GO:0020039) and apical part of the cell (GO:0045177). A substantial proportion corresponded to alveolin domain–containing filaments and inner membrane complex (IMC) transcripts. Several genes encoding ApiAP2 transcription factors were either up or downregulated (Figure 3), potentially affecting the expression of stage-specifically regulated genes.

Sixty-one transcripts coding for SRS proteins were upregulated following BKI-1708 treatment, and 19 were downregulated (see Supplementary Figure S1). Among those, transcripts associated with enteroepithelial stages (EES), thus sexual reproduction [35,36], were also differentially regulated, but were notably more prevalent among the upregulated transcripts, ranging from the early merogony and gametocytogenesis to the late sexual stages. Transcripts associated with tachyzoites such as SAG1, LDH and ENO2 were mainly found to be expressed at lower levels compared to untreated tachyzoites, while several bradyzoite-associated transcripts were upregulated (Figure S2).

Genes encoding key factors involved in invasion and egress were also found to be transcribed at lower levels, including microneme, rhoptry, and dense granule proteins, as well as gliding-associated proteins. Notably, transcript levels of the actin-binding protein toxofilin and perforin-like protein 1 (PLP1) were also significantly reduced upon treatment. The observed downregulation of ribosomal genes and transcripts of eukaryotic initiation factors indicates a general reduction in translational capacity. Redox-associated transcripts, including thioredoxin and thioredoxin-like proteins also showed decreased levels. Importantly, MAPKL-1 mRNA, coding for one of the two reported molecular targets of several BKIs including BKI-1708, was among the downregulated transcripts.

In BKI-1708-treated *T. gondi* tachyzoites, upregulated transcripts also contained a high proportion of hypothetical proteins, comprising almost half of the transcripts (47%). GO analysis did not identify any distinct pathways. Messenger RNA coding for IMC3 exhibited upregulated expression, and transcripts coding for markers associated with bradyzoite differentiation were increased, including enolase 1, bradyzoite formation deficient 2 (BFD2), and the serin palmytoil transferases 1 and 2 (SPT1, SPT2) involved in rhoptry biogenesis and cyst formation. Upregulation of perforin-like protein 2 was also observed. Several calcium-dependent protein kinase transcripts, including CDPK2B, CDPK4B, and CDPK7A, were increased, along with transcripts encoding dense granule proteins (including merozoite-specific GRA11A and GRA11B) and a subset of microneme and rhoptry components. Furthermore, four oocyst wall protein transcripts were also found in treated parasites, pointing to a disruption of stage-specific gene expression programs.

#### 3.2.2. Correlation of Transcript and Protein Abundance Changes in *T. gondii* upon BKI-1708 Treatment

**Figure 4:**
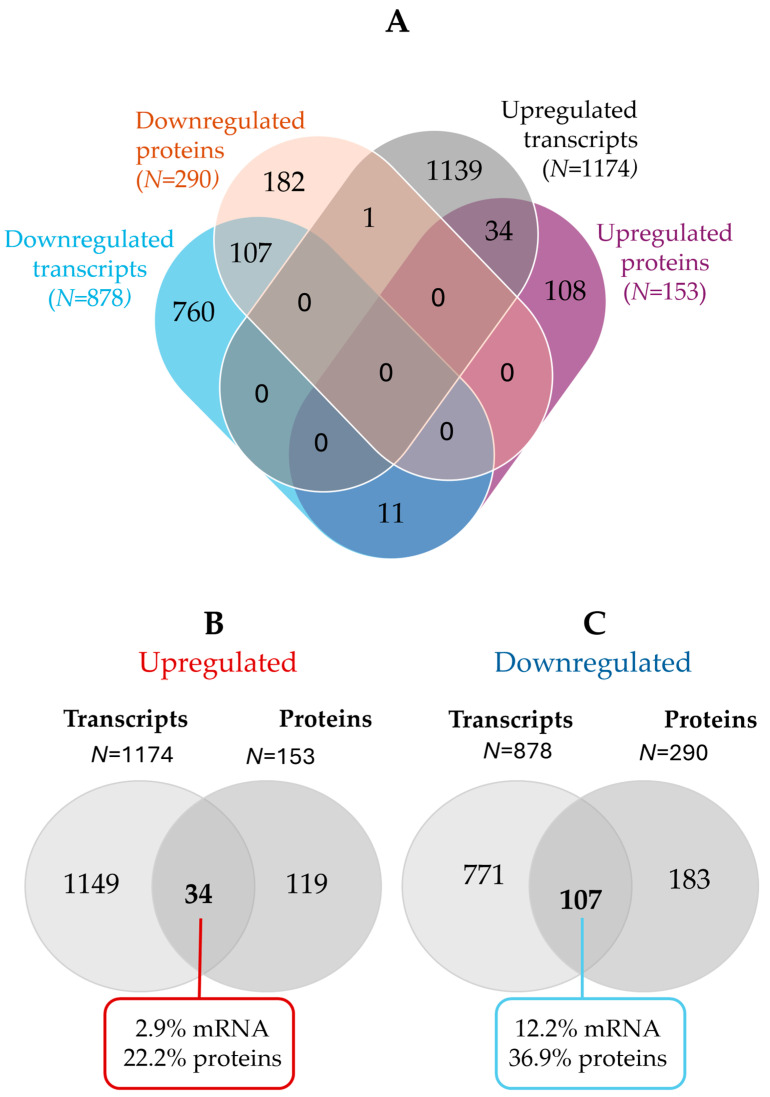
Venn diagrams showing the number of genes significantly regulated following BKI-1708 treatment, at the protein level, the transcript level, and the overlap between both datasets compared to untreated tachyzoites (**A**). (**B**,**C**) shows the numbers and percentages of concordantly regulated genes detected in both transcriptomic and proteomic datasets for upregulated and downregulated transcripts/proteins, respectively. Percentages indicate the proportion of genes showing corresponding regulation relative to the total number of up or downregulated transcripts (% mRNA) or proteins (% proteins); *N* indicates the total number of transcripts/proteins detected.

Analysis of the combined transcriptomic and previously published proteomic data [32] revealed a subset of proteins displaying concordant regulation upon BKI-1708 treatment (Figure 4A). The full data can be accessed in Supplementary Table S4.

**Figure 5:**
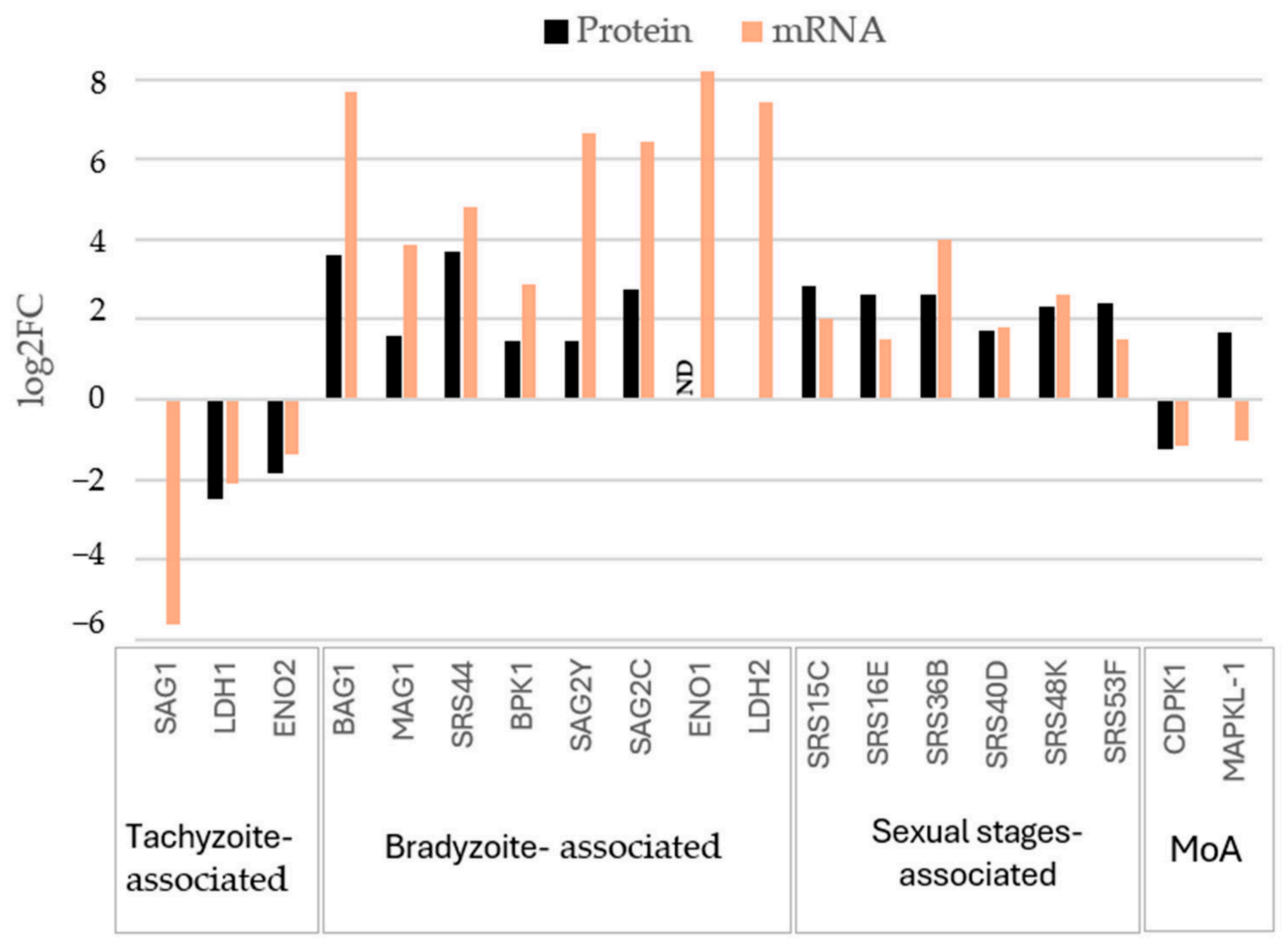
Summary of changes in protein abundance and mRNA levels of selected genes associated with the tachyzoite, bradyzoite, and sexual stages of *T. gondii* upon treatment with BKI-1708, as well as CDPK1 and MAPKL1 involved in the mode of action of the compound. MoA = mode of action; ND = not detected; Log2FC—log2-transformed fold changes. Fold change in mRNA levels derived from DESeq2-normalized RNA-seq read counts; those from proteins originate from [32].

A smaller set comprising 34 proteins and respective transcripts expressed at higher abundance (Figure 4B) included several bradyzoite markers and EES-associated SRS-family proteins, proteins linked to redox metabolism and secretary organelle-related proteins. In the group of 107 transcripts with simultaneous downregulation of protein and transcript expression (Figure 4C), most coded for ribosomal proteins and proteins with catalytic activity, followed by RNA-binding proteins, as well as CDPK1, toxofilin, eukaryotic initiation factor-2, microneme proteins and rhoptry kinases, including ROP18. Figure 5 summarizes the correlation between protein abundance and mRNA levels for some genes of interest in treated *T. gondii*, including tachyzoite-, bradyzoite- and sexual-stage-associated genes, as well as those coding for CDPK1 and MAPKL1.

#### 3.2.3. Inverse Correlation Between Transcript and Protein Expression Levels in *T. gondii* Under BKI-1708 Treatment

Discrepancies between mRNA and protein expression upon BKI treatment were identified (Figure 4A). Specifically, one hypothetical protein (TGME49_315270) was uniquely found at lower abundance in treated parasites, but mRNA levels were higher compared to controls. In contrast, 11 other proteins were found to be expressed at higher levels in treated parasites, but the corresponding transcript levels were reduced. This set comprised several hypothetical proteins as well as characterized candidates that included one of the canonical targets, MAPKL-1. The list of proteins with increased protein and reduced transcript abundances is shown in Table 2.

### 3.3. Transcriptomic Profiling Reveals BKI-1708–Induced Changes in T. gondii-Infected and Non-Infected Human Fibroblasts

RNA sequencing of non-infected and *T. gondii*-infected HFF identified 42,427 and 42,852 transcripts, respectively. Differential expression analysis revealed that 1174 (2.7%) and 2561 transcripts (5.9%) were upregulated in non-infected and infected HFF, respectively, whereas 923 (2.2%) and 1998 transcripts (4.6%) were downregulated (Table 1). This indicates that BKI-1708 induces changes upon the host cell steady-state transcriptome (Figure 2). Overlap of identified transcripts is shown in Supplementary Figure S3. To identify host cellular pathways affected by BKI-1708 treatment, differentially expressed genes were subjected to KEGG pathway enrichment analysis. The complete KEGG enrichment results can be accessed in Figure S4. Overall, KEGG pathway enrichment analysis revealed distinct infection-dependent transcriptional responses of HFF to BKI-1708 treatment.

In non-infected HFF, downregulated pathways were predominantly associated with transcripts coding for extracellular matrix (ECM) organization and cytoskeletal dynamics, including ECM–receptor interaction, focal adhesion, and regulation of the actin cytoskeleton, alongside cardiac-related pathways and PI3K–Akt signaling. Conversely, transcripts expressed at higher levels in non-infected cells were enriched for metabolic and detoxification processes, such as steroid biosynthesis, cholesterol metabolism, ABC transporters, and xenobiotic metabolism, indicating a shift towards metabolic adaptation. Upregulation of genes involved in xenobiotic metabolism was observed in BKI-treated non-infected cells, particularly within the cytochrome P450 pathway (hsa00980). This included increased expression of mRNA coding for alcohol dehydrogenases (ADH1B, ADH1C), phase I metabolism enzyme cytochrome P450 subfamily B1 (CYP1B1), and multiple phase II detoxification components such as aldo ketoreductase family member 1 (AKR1C1), dihydrodiol dehydrogenase (DHDH), and glutathione S-transferases (GSTM1, GSTM2, GSTM5). In parallel, genes associated with the ABC transporter pathway (hsa02010) were also upregulated, including lipid transporters (ATP-binding cassette, subfamily A, member 6, 9 and 10; ABCA6, ABCA9, ABCA10), multidrug resistance-associated transporters ABCB5 and ABCC6, the efflux pump ABCG2, and the ion channel cystic fibrosis transmembrane regulator CFTR.

In infected HFF, BKI-1708 treatment induced a different response. Downregulated pathways were strongly enriched in mRNA coding for proteins involved in cell cycle progression, DNA replication, and repair mechanisms, as well as immune-related signaling pathways including tumor necrosis factor (TNF) and interleukin 17 (IL-17) signaling, reflecting suppression of proliferative and inflammatory responses. Components of the Fanconi anemia and homologous recombination pathways, breast cancer gene 1 (BRCA1), the recombinase RAD51, Bloom syndrome protein (BLM), Fanconi amenia group D2 protein (FANCD2), and x-ray repair cross-complementing 2/3 (XRCC2/3) were also suppressed, likely reflecting impaired DNA damage repair. Additionally, transcripts involved in nucleotide metabolism, including those coding for ribonucleoside diphosphate reductase (RRM1/2), thymidylate synthase (TYMS), adenylate cyclase 6 (AK6), and calcium and TNF signaling (mitogen-activated protein kinase 8 (MAPK8), IL6, colony stimulating factor 2 (CSF2), and the chemokine CXCL2), were downregulated. In contrast, transcripts were upregulated upon infection and treatment coding for factors involved in host defense and degradation processes, including lysosome function, antigen processing and presentation, complement and coagulation cascades, and glycosaminoglycan degradation, alongside pathways linked to pathogen interaction such as *Staphylococcus aureus* infection and viral infection signatures. Transcripts coding for components of the complement system (C3, C4A/B, CFB, CFD) and interferon-responsive factors (IRF7, IRF99 and key signal transducers in the JAK-STAT pathway (STAT1, STAT2) were elevated. Structural and cytoskeletal remodeling transcripts, such as integrin subunit alpha 11 and beta 4 (ITGA11, ITGB4), laminin subunits 2–5 (LAMA2–5), cartilage oligomeric matrix protein (COMP) and collagen 1A2 (COL1A2), were also enriched.

#### 3.3.1. Transcriptional Responses to BKI-1708 Commonly Found in Infected and Non-Infected HFF

**Figure 6:**
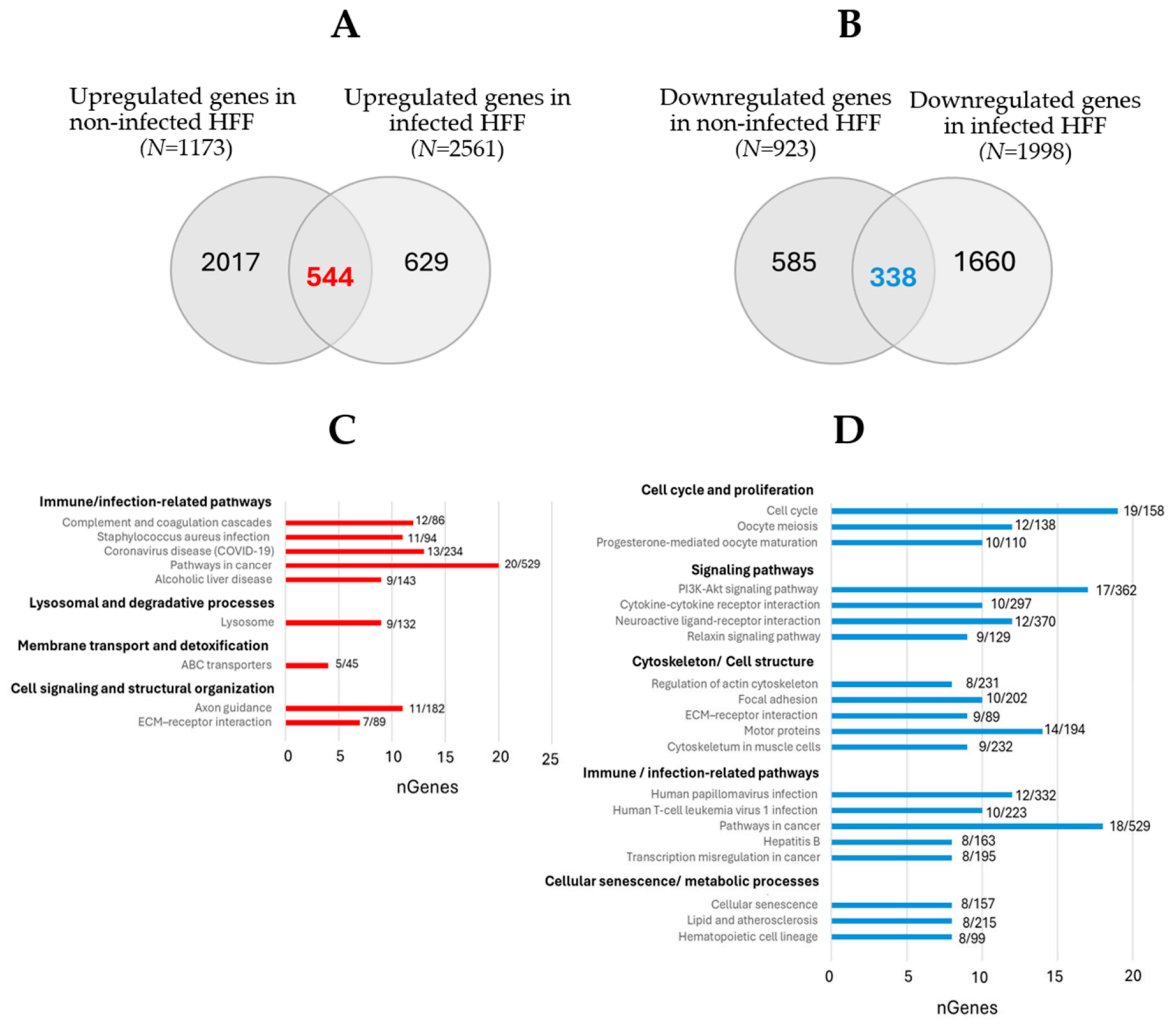
Venn diagrams showing the overlap of upregulated (**A**) and downregulated (**B**) transcripts in non-infected and infected host cells upon BKI-1708 treatment, representing common responses to BKI-treatment regardless of infection status. Panels (**C**,**D**) show the summary of KEGG pathways affected by BKI-1708 treatment in infected and non-infected HFF, as identified from enrichment analyses of upregulated and downregulated transcripts. Each pathway shows the number of affected genes (nGenes) in relation to the total number of genes of the respective pathway.

While the transcriptional responses of infected and non-infected HFF induced by BKI-1708 were largely distinct, a subset of differentially expressed genes was common in the two conditions (Figure 6A,B). Specifically, 544 gene transcripts were consistently upregulated, and 338 transcripts were consistently downregulated in both infected and non-infected cells, indicating a conserved host response to BKI-1708 independent of infection status. To investigate the functional relevance of the shared transcriptional response, KEGG pathway enrichment analysis was performed on these commonly regulated genes (Figure 6C,D) and revealed distinct functional trends. Upregulated genes were primarily associated with complement and infection-related pathways, lysosomal processes, ABC transporters, and extracellular matrix (ECM)–receptor interactions. Downregulated genes were significantly enriched in pathways related to cell cycle progression, cytokine–cytokine receptor interactions, phosphoinositide 3 kinase-protein kinase B (PI3K-Akt) signaling pathway, cytoskeletal organization, and cellular senescence.

#### 3.3.2. Host Gene Expression Changes Induced by *T. gondii* Infection in BKI-1708-Treated Cells

A subset of transcripts showed opposing expression patterns depending on infection status. In total, 73 transcripts were upregulated in infected BKI-1708-treated cells while being downregulated in respective non-infected cells, whereas 65 transcripts were downregulated upon infection but upregulated in non-infected cells (Figure S4, Supplementary Table S3). KEGG analysis of the 73 upregulated genes in infected HFF and downregulated in non-infected HFF resulted in 73 IDs mapped to 64 human genes. Five transcripts were associated with the cytoskeleton or extracellular matrix, namely thrombospondin (THBS), synaptopodin 2 (SYNPO2), PDLIM connecting both cytosolic and membrane proteins to actin filaments, myosin-binding protein H (MYBPH) and the fibroblast marker integrin-alpha1 (ITGA).

No significantly enriched pathway could be identified in the set of 65 transcripts that were downregulated in infected but upregulated in non-infected HFF. Among these, several regulators of immune responses were identified, including the interferon regulatory factor (IRF4), the pro-inflammatory cytokines interleukin 1A and 23A (IL1A, IL23A) and the hepatocyte nuclear factor 3-alpha A1 (FOXA1)—as well as transcripts coding for proteins induced by cytokine signaling and immune modulation, such as cytokine-induced SH containing protein (CISH), and natural killer cell cytotoxicity receptor 3 ligand 1 (NCR3LG1), a protein that acts as ligand for the NK cell receptor 3, mostly expressed in tumor cells.

## 4. Discussion

BKI-1708 treatment induced significant transcriptional reprogramming in *T. gondii*, with approximately 25% of all genes showing differential, and mostly upregulated, expression. In contrast, HFF host cells exhibited comparatively limited transcriptome changes. Less than 5% of genes were affected in non-infected HFF, while around 10% were affected in *T. gondii*-infected cells. The stronger response in infected host cells suggests that the observed transcriptional alterations are partly driven by the *T. gondii* infection context. This indicates a combined effect of drug treatment and parasite presence.

### 4.1. BKI-1708 Induced Transcriptome Changes in T. gondii

Exposure of *T. gondii* tachyzoites to BKI-1708 caused a significant disruption in the expression of transcripts involved in cytoskeletal organization, invasion machinery, and stage-specific gene expression. The upregulated gene set lacked clear functional enrichment, but most of them were ApiAP2 transcription factors, SRS proteins (including transcripts of genes associated with enteroepithelial stages—sexual reproduction), and multiple bradyzoite-associated markers. The upregulation and downregulation of ApiAP2 transcription factors indicate a transcriptional dysregulation upon BKI-1708 treatment. Overall, the functional roles of many of the identified transcription factors remain unclear. However, previous studies have shown that the transcription factor AP2IV-4, whose transcripts are found at lower abundance upon BKI-1708 exposure, acts as a repressor of bradyzoite-specific gene expression. It directly silences bradyzoite mRNA and protein expression during the acute tachyzoite stage [37]. TgAP2IX-5 (downregulated upon BKI-1708 exposure) has been shown to bind to the promoter of TgAP2XII-9 and AP2III-2 (both also downregulated upon BKI exposure), regulating the expression of a large number of IMC and apical complex genes, synchronizing cellular and organelle replication during *T. gondii* asexual division [38,39]. Moreover, transcripts for AP2XII-2, implicated in gene regulatory pathways controlling *T. gondii* sexual development, were found at lower levels in treated parasites compared to untreated tachyzoites [40]. Depletion of AP2XII-2 resulted in delayed S-phase progression, slowed parasite replication, and disruption of transcriptional repression, allowing the expression of the reported merozoite-specific AP2X-10 (upregulated under BKI-1708 treatment) and oocyst wall-associated genes in tachyzoites [41]. Moreover, a subset of transcripts for AP2 factors reportedly enriched in *T. gondii* gametes, such as AP2III-4, AP2X-10 and AP2IX-3, were upregulated in BKI-1708-treated parasites, while AP2III-1 (also linked to sexual stages) was downregulated [42]. The simultaneous expression of transcripts coding for bradyzoite and tachyzoite markers, and of mRNA coding for proteins normally expressed in enteroepithelial stages and for oocyst wall proteins, further suggests a disruption of canonical developmental programs.

The observed upregulation of bradyzoite-associated genes and downregulation of some tachyzoite markers, along with the downregulation of 51 ribosomal genes, reflect a transition towards a state with reduced metabolic activity. Aligned with this, transcripts for apical complex–related proteins and proteasome components were found at lower abundance upon treatment. In parallel, downregulated transcripts were enriched for mRNA coding for IMC and pellicle-associated components, including alveolin-domain proteins, and important factors involved in motility, invasion, and egress. As an obligate intracellular parasite, successful and efficient host cell invasion is of paramount importance for *T. gondii*. To achieve this, the parasite relies on a coordinated secretion of microneme and rhoptry neck proteins (RONs) [43,44]. Initially, apical membrane antigen 1 (AMA1) is released from micronemes onto the parasite surface [45,46]. However, upon treatment with BKI-1708, the transcripts of 37 rhoptry proteins, including RON2, RON4, RON5, and RON8, which form a preformed complex that binds the previously secreted AMA1 microneme protein [45,47], were expressed at lower abundance compared to untreated parasite controls. These proteins collectively form the moving junction complex, which is essential for host cell invasion [46,48]. Interestingly, upon BKI-1708 treatment, transcripts coding for glideosome-associated or gliding-associated proteins were also expressed at lower abundance compared to untreated tachyzoites.

GO analysis of downregulated genes revealed significant enrichment of pathways related to the inner membrane pellicle complex (GO:0070258), pellicle (GO:0020039), and the apical part of the cell (GO:0045177). These pathways include 16 transcripts encoding IMC proteins and 12 transcripts coding for alveolin domain–containing filaments. Notably, the only IMC-related gene exhibiting upregulated transcript levels upon BKI-1708 treatment was the one coding for IMC3, a protein that is preferentially associated with the budding daughter cytoskeleton [49].

Transcriptomic analysis revealed that BKI-1708 treatment significantly decreased the abundance of transcripts coding for toxofilin and perforin-like protein (PLP1). Toxofilin is involved in manipulation of the actin cytoskeleton and is important for host cell invasion [50,51]. PLP1-deficient parasites exhibit impaired parasite egress and reduced parasite virulence, and PLP1 was linked to a lethal inflammatory immune response during acute infection with a virulent strain of the parasite [52]. This was accompanied by decreased expression of transcripts related to translation and redox processes, including thioredoxin 1 and oxidoreductase, suggesting an overall decline in parasite fitness.

Two BKI-drug targets have been defined and validated, namely CDPK1 and MAPKL1. For both, the expression of the corresponding transcripts was downregulated upon BKI-1708 treatment. However, several calcium-dependent protein kinase transcripts, including CDPK2B, CDPK4B, and CDPK7A, were upregulated, indicating a compensatory remodeling of calcium-dependent kinase signaling pathways. As a coordinated increase in both mRNA and protein levels signifies canonical activation, and conversely, a coordinated decrease in mRNA and protein levels indicates canonical repression, the correlation between mRNA levels and protein abundance in treated tachyzoites was investigated. Upon BKI-1708 treatment, both mRNA and protein levels of CDPK1 were reduced. This coordinated downregulation of the transcript and protein could be attributed to (i) direct transcriptional repression, (ii) mRNA destabilization, or (iii) feedback mechanisms triggered by inhibition of CDPK1 activity. Unlike genes exhibiting discordant mRNA–protein patterns, CDPK1 appears to be sensitive to both transcriptional and post-transcriptional control, which is consistent with its essential role in parasite survival and growth [53,54]. Remarkably, MAPKL-1 mRNA levels were decreased in baryzoites, but exhibited increased protein abundance. Possible explanations could include (i) enhanced translation efficiency, (ii) increased protein stability/reduced degradation, or (iii) protein sequestration [55]. In the context of BKI-1708, given its RNA-binding properties and the associated downregulation of ribosomal proteins, overall translation is likely impaired. This suggests that MAPKL-1 protein accumulation may result from selective translation of specific transcripts or exceptional protein stability, allowing it to persist even when global protein synthesis is suppressed. It is plausible that altered RNA–protein interactions promote sequestration of specific proteins into complexes, a mechanism reminiscent of translationally repressed mRNA storage observed in apicomplexan parasites [56,57]. This process involves the storage of mRNAs in a non-functional state within cytoplasmic stress granules and ribonucleoprotein complexes. In previous studies, electron microscopy detected increased numbers of cytoplasmic granules upon treatment of *T. gondii* tachyzoites with a combination of the related BKI-1748 and artemisone [58], as well as in *Neospora caninum* and *Besnoitia besnoiti* baryzoites treated with BKI-1708 [32]. However, this discordant pattern, characterized by low mRNA levels but high protein abundance, was observed in ten other gene products, including chromatin components (H2AZ, CENH3), cytoskeletal and signaling proteins (IMC12, putative Calmodulin), surface antigens (SRS30A), a mitochondrial ATP synthase-associated protein, and several uncharacterized hypothetical proteins.

Expression levels of stage-specific marker proteins and their corresponding transcripts displayed a high degree of correlation: tachyzoite-marker proteins and corresponding transcripts were expressed at lower levels, whereas bradyzoite markers and respective mRNA levels were increased upon treatment. Discrepancies between mRNA and protein levels upon BKI-1708 treatment, such as for the tachyzoite marker SAG1 (with decreased transcript levels without a corresponding change in protein abundance at the timepoint of analysis), could indicate that the protein is highly stable and resistant to turnover, or that translational buffering maintains steady protein levels despite lower mRNA. In contrast, LDH2, a classical bradyzoite marker, displayed increased transcript levels, yet protein abundance remained unchanged, indicating potential post-transcriptional regulation, such as limited translation efficiency or rapid protein degradation. Notably, one hypothetical protein exhibited increased mRNA levels but decreased protein abundance, suggesting post-transcriptional regulation or enhanced protein turnover.

### 4.2. Transcriptomic Profiling of Host Cells Reveals Distinct Patterns of Transcriptional Changes upon BKI-1708 Treatment, Depending on the Infection Status

Transcriptomic profiling of host cells revealed that BKI-1708 treatment induced distinct patterns of transcriptional changes, depending on the infection status. In infected cells, pathways associated with lysosomal degradation, antigen processing, and immune activation were significantly upregulated. Transcripts of lysosomal genes, including those coding for LAMP1, CTSD, CTSL, HEXA, and HEXB, indicated enhanced degradative capacity. Simultaneously, increased expression of HLA-A, HLA-B, HLA-C, HLA-DMA, HLA-DOA, and HLA-DPA1, along with TAP1 and TAPBP transcripts, reflected activation of both MHC class I and II antigen presentation pathways. Transcripts coding for components of the complement system (C3, C4A, C4B, CFB, and CFD) and interferon-responsive regulators (IRF7, IRF9, STAT1, and STAT2) were also elevated. Additionally, transcripts of genes involved in structural organization and cytoskeletal remodeling were also transcribed at higher levels, suggesting coordinated host cell architectural changes during infection. In contrast, pathways involved in DNA replication, cell cycle progression, and DNA repair were broadly downregulated, with reduced expression of key regulators (e.g., MCM2–7, CDC6, CDK1, CCNA2, CCNB1/2, PLK1), indicating suppressed host proliferation. Parallel downregulation of nucleotide metabolism genes (RRM1/2, TYMS, AK6) and signaling components (MAPK8, IL6, CSF2, CXCL2) suggests decreased metabolic activity and attenuated inflammatory responses, collectively supporting a host environment conducive to parasite survival.

In non-infected HFF treated with BKI-1708, genes involved in xenobiotic metabolism, particularly within the cytochrome P450 pathway (hsa00980), were upregulated. This included transcripts coding for lipid transporters (ABCA6, ABCA9, ABCA10), multidrug resistance-associated transporters (ABCB5, ABCC6), the efflux pump ABCG2, and the ion channel CFTR. These changes suggest a coordinated cellular response to BKI exposure, characterized by enhanced metabolic processing and active efflux of xenobiotic compounds. Conversely, the downregulation of transcripts of integrin components (ITGB1, ITGA6) and ECM ligands (LAMC2, THBS1, SPP1) indicates impaired cell–matrix signaling and concomitant suppression of PI3K–Akt pathway nodes, including EGF, MET, and IL6. This is accompanied by reduced expression of key cell cycle regulators, collectively suggesting diminished adhesion-dependent signaling, survival pathways, and proliferative capacity.

Interestingly, several pathways were commonly affected in both infected and non-infected HFF, indicating a conserved host response to treatment independent of infection. Upregulated genes were mainly enriched in complement and infection-related pathways, lysosomal processes, ABC transporters, and ECM–receptor interactions, suggesting stress adaptation and cellular remodeling. In contrast, downregulated genes were associated with cell cycle progression, cytokine signaling, PI3K–Akt signaling, cytoskeletal organization, and cellular senescence, suggesting a degree of suppression of proliferation, signaling, and structural dynamics in treated cells.

A subset of genes exhibited opposing regulation depending on infection status, indicating that BKI-1708 triggers different host responses, reflecting host–parasite interaction dynamics. Transcripts of genes upregulated in infected but downregulated in non-infected cells were primarily linked to cytoskeletal organization, suggesting parasite-specific remodeling processes that may support intracellular survival or reflect host attempts to control infection [7,46]. Conversely, genes downregulated in infected but upregulated in non-infected cells included key regulators of cytokine signaling and transcription, pointing to a differential modulation of immune responses that could either result from parasite-driven immune evasion or altered host signaling under treatment [59]. It is known that *T. gondii* actively modulates host transcription programs [57,60,61]. The observed inversion of gene expression between infected and non-infected cells reflects this host–pathogen interaction, in which BKI-1708-induced perturbations intersect with these infection-driven signaling networks.

Seven proteins whose correspondent transcript levels were reduced upon BKI-1708 treatment have been identified earlier by differential affinity chromatography as BKI-1708-binding proteins [33]. The set included three hypothetical proteins (TGME49_209420, TGME49_205320; TGME49_250115), an RNA recognition motif-containing protein (TGME49_205180), dense granule protein GRA62 (TGME49_215360), tubulin-tyrosine ligase family protein (TGME49_244500), thioredoxin-like associated protein TLAP4 (TGME49_201760) and perforin-like protein PLP1 (TGME49_204130). GRA 62 is located at the parasitophorous vacuole membrane [62], while PLP1 is essential for parasite egress and the induction of host inflammatory responses [52]. *T. gondii* encodes two perforin-like proteins: TgPLP1 and TgPLP2 [63]. Unlike PLP1, PLP2 has not been extensively characterized; however, it has been reported not be expressed in the tachyzoite stage [64]. Upon BKI-1708 exposure, PLP2 transcripts were detected at higher levels compared to controls, but the corresponding protein was not detected in the proteome of BKI-1708 induced baryzoites [32]. The functions of proteins from the tubulin-tyrosine ligase (TTL) family in *T. gondii* are not well defined; however, it is known that TTL enzymes regulate tubulin tyrosination/detyrosination, shaping microtubule stability and cytoskeletal organization [65]. Studies in the kinetoplastid *Trypanosoma brucei* showed that TTL disruption altered microtubule modifications, flagellar motility, and parasite physiology [66]. By analogy, BKI-1708 binding to TTL in *T. gondii* may similarly perturb microtubule post-translational modifications, affecting motility, structural organization, or intracellular processes. Finally, TLAP4 is a redundant stabilizer of cortical microtubules in daughter parasites, ensuring the cytoskeleton remains intact even when primary stabilizers are compromised during replication [67]. This pattern of high affinity to the compound and low transcripts under treatment suggests that compound binding may trigger feedback mechanisms or sequestration events that reduce transcript abundance, or alternatively, that protein stabilization by BKI-1708 permits the cell to lower transcription while maintaining functional protein levels.

## 5. Conclusions

In summary, BKI-1708 treatment exerts observable effects on the host cell transcriptome. Exposure of *T. gondii* tachyzoites to BKI-1708 reduces the transcription of genes linked to cytoskeletal organization, invasion machinery, and translation. Concurrently, the expression of bradyzoite-associated transcripts is increased, while the expression of tachyzoite markers, ribosomal, and redox-related transcripts is inhibited. This suggests a shift towards a less metabolically active state, resulting in reduced fitness and invasion potential. In contrast, BKI-1708 induces limited transcriptional changes in host cells, affecting a specific set of pathways without broadly compromising essential cellular functions. Overall, the treatment elicits overlapping but context-specific responses: uninfected cells predominantly activate metabolic and detoxification programs, while *T. gondii* infected cells enhance immune, lysosomal, and glycan degradation pathways. Collectively, these findings support the potential of BKI-1708 as a selective anti-parasitic compound that interferes with parasite biology while preserving host cell integrity.

## Figures and Tables

**Table 1:** Summary of differential gene expression across treatment conditions. Total detected genes and numbers of significantly overexpressed and downregulated genes are shown for *T. gondii*, non-infected human foreskin fibroblasts (HFF), and *T. gondii*-infected HFF. Differential expression (DE) was defined according to the thresholds described in Section 2.

	*T. gondii*	Non-Infected HFF	Infected HFF
Total number of annotated genes	8140	42,427	42,852
Number of genes upregulated at transcript level (≥2 fold)	1174 (14.4%)	1173 (2.7%)	2561 (5.9%)
Number of genes downregulated at transcript level (≤2 fold)	878 (10.7%)	923 (2.2%)	1998 (4.6%)

**Table 2:** Proteins and corresponding mRNA levels with discordant regulation upon BKI-1708 treatment. The proteins are listed according to their decreasing rAbu values, and rAbu values were published earlier in [32]. The relative abundances (rAbu) are based on iBAQ (intensity-based absolute quantitation), summed up to a total of 1,000,000 for each sample.

Messenger RNA Levels Decreased and Protein Levels Increased Upon BKI-1708 Treatment			
Toxo ID	Annotation	mRNA Fold Change	Protein Fold Change
TGME49_300200	Histone H2AZ	0.33	2.35
TGME49_225410	Histone H3 centromeric CENH3	0.47	3.10
TGME49_249240	Calmodulin, putative	0.41	2.79
TGME49_248700	Alveolin domain containing intermediate filament IMC12	0.45	3.78
TGME49_226570	Hypothetical protein	0.27	2.31
TGME49_273130	SAG-related sequence SRS30A	0.36	2.30
TGME49_236950	Hypothetical protein	0.28	2.70
TGME49_270360	ATP synthase-associated protein	0.46	2.87
TGME49_312570	CMG kinase, MAPK family (ERK) MAPK-1	0.47	3.14
TGME49_229220	Hypothetical protein	0.20	2.53
TGME49_219730	Hypothetical protein	0.46	2.99

## Data Availability

Raw read files of sequences are deposited at the European Nucleotide Archives (ENA) (http://www.ebi.ac.uk/ena) (study PRJEB111857) and will be openly accessible from 23.07.2026. The original contributions presented in this study are included in the article/Supplementary Materials. Further inquiries can be directed to the corresponding authors.

## Supplementary Materials

The following supporting information can be downloaded at: https://www.mdpi.com/article/10.3390/cells15151324/s1, Figure S1: Structural changes induced by BKI-1708 treatment upon *T. gondii* tachyzoites visualized by SEM and TEM; Figure S2: Stage-specific SRS-family genes up/downregulated upon BKI-1708 treatment; Figure S3: Venn diagram showing the specific and overlapping distribution of host genes expressed in *T. gondii*-infected and non-infected HFF; Figure S4: Pathways enriched among downregulated and upregulated genes in uninfected and *T. gondii*-infected HFF treated with BKI-1708; Table S1: Dataset of up and downregulated transcripts in uninfected and *T. gondii*-infected HFF treated with BKI-1708; Table S2: Dataset of up and downregulated transcripts *T. gondii* treated with BKI-1708; Table S3: List of up and downregulated SRS transcripts of *T. gondii* treated with BKI-1708; Table S4: List of gene IDs corresponding to the intersection of differentially expressed genes identified in *Toxoplasma gondii* in response to BKI-1708 treatment.
