# Supplementary material for: Transcriptional Responses to Bumped Kinase Inhibitor BKI-1708 in Toxoplasma gondii and Human Fibroblasts
Source: Cells. 2026 Jul 24;15(15):1324. doi: 10.3390/cells15151324 (PMC13465175; doi:10.3390/cells15151324)
Supplement: Supplementary file 1 [file cells-15-01324-s001.zip › SuppFigures.pdf]

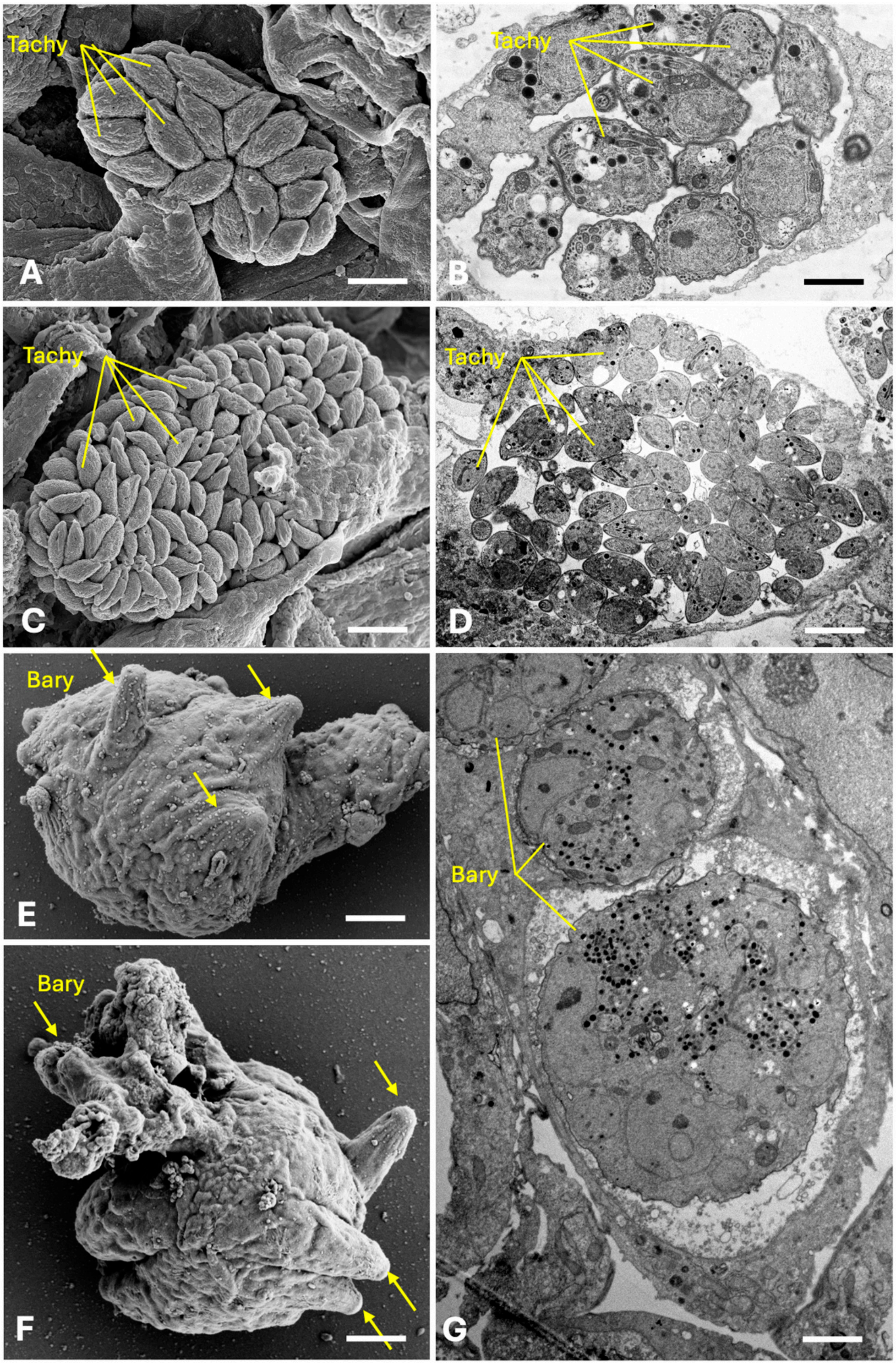

Figure S1. Structural changes induced by BKI-1708 treatment upon *T. gondii* tachyzoites visualized by SEM and TEM. SEM (**A, C, E, F**) and TEM (**B, D, G**) of *T. gondii* grown in HFF. Samples were fixed and processed as described in [27]. (**A-D**) show tachyzoites (Tachy) of non-treated cultures maintained during 48 h. (**E-G** are examples of baryzoites (Bary) formed after exposure to BKI-1708 (2.5 μM, 5 days). Bar in **A** = 4 μm, **B** = 1.3 μm, **C** = 3 μm, **D** = 1.9 μm; 2.6 μm. **E** = 0.9 μm, **F** = 0.9 μm, **G** = 2 μm.

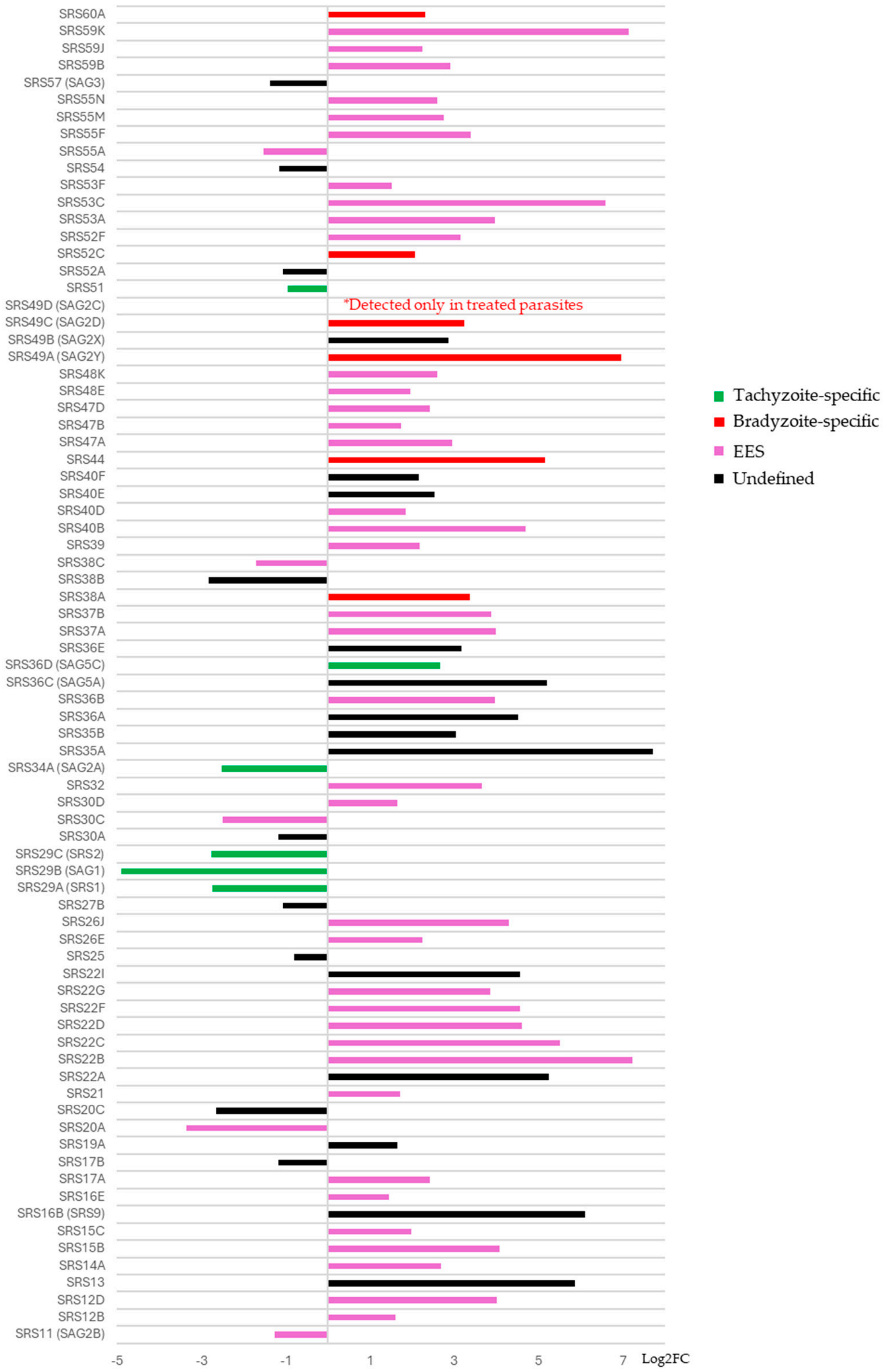

Figure S2. Stage-specific SRS-familiy genes are up- or downregulated upon BKI-1708 treatment. EES – entero-epithelial stages. Log2FC – log2 transformed fold changes. Fold change in mRNA levels derived from DESeq2-normalized RNA-seq read counts.

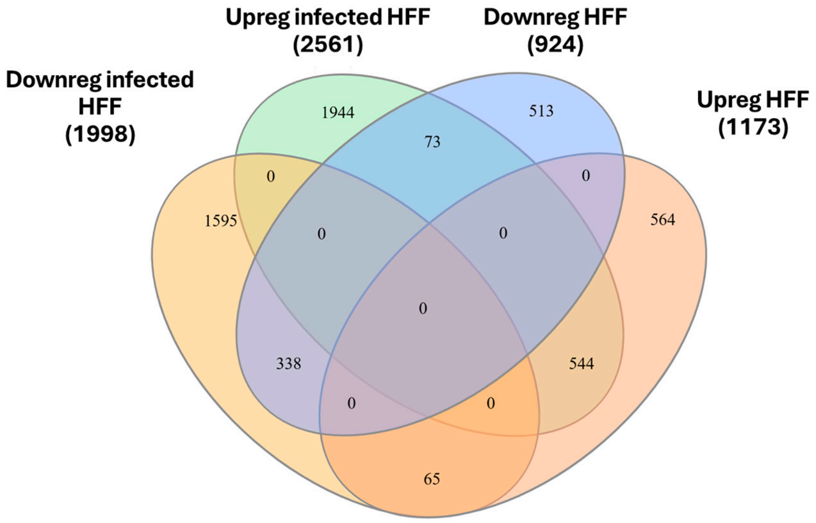

Figure S3. Venn diagram showing the specific and overlapping distribution of host genes differentially expressed in *T. gondii* infected and non-infected cells. Numbers in each section indicate the count of genes transcripts shared between the respective datasets.

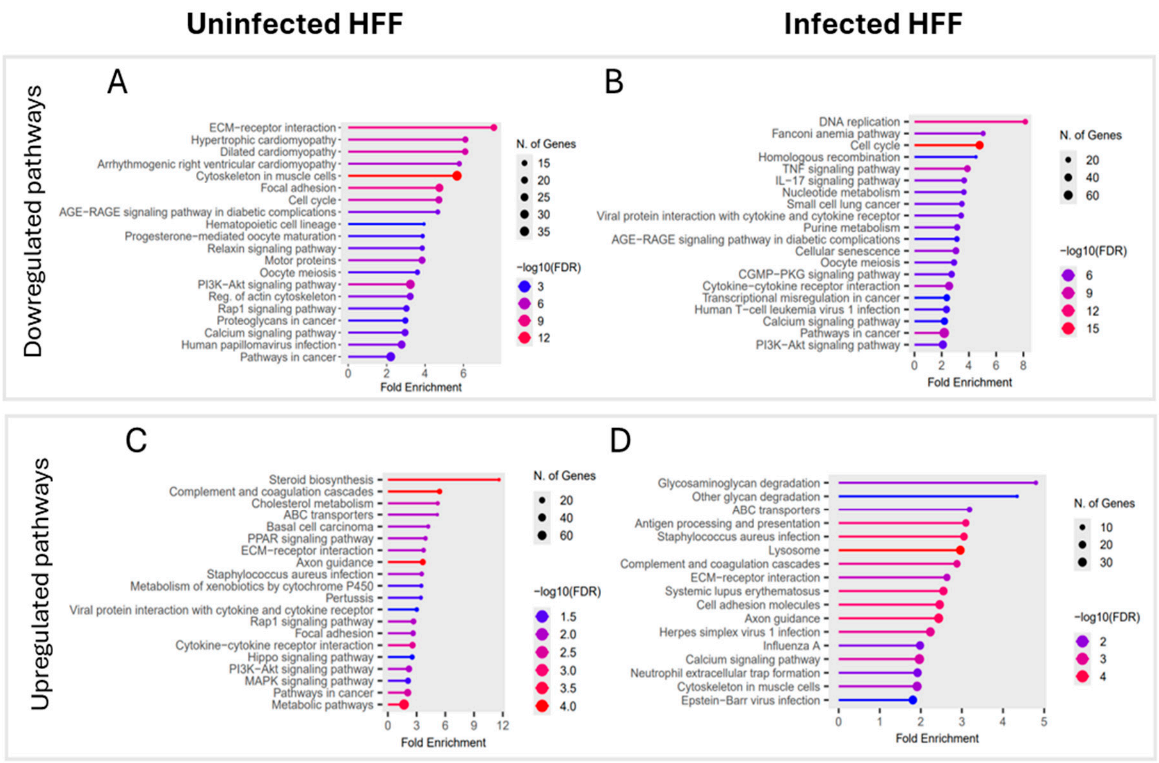

Figure S4. Pathways enriched amongst downregulated (**A, B**) and upregulated (**C, D**) genes in uninfected (**A, C**) and *T. gondii*-infected (**B, D**) human foreskin fibroblasts (HFFs) are shown. Pathways are ranked by fold enrichment (x-axis), with the number of associated genes indicated by circle size. Circle color represents the statistical significance of enrichment as –log10(FDR), with darker/more intense colors indicating higher significance.
